# Effects of Dietary Tea Polyphenols on the Growth, Antioxidant Status, Immune Function, and Intestinal Microbiota of Largemouth Bass (*Micropterus salmoides*)

**DOI:** 10.3390/ani15020222

**Published:** 2025-01-15

**Authors:** Zixin Yang, Qiuwen Su, Jiafa Yang, Zhijun Li, Shanren Lan, Xu Jia, Paihuai Ouyang, Huijuan Tang

**Affiliations:** 1 College of Marine Sciences, South China Agricultural University, Guangzhou 510642, China; 2 Guangdong Weilai Biotechnology Co., Ltd., Guangzhou 511400, China

**Keywords:** tantibiotic substitutes, carnivorous freshwater fish, antioxidant capacity, immune response, intestinal microbiota

## Abstract

This research aimed to explore the impact of tea polyphenol (TP) supplementation on the development, antioxidant properties, immune responses, and gut wellness in largemouth bass (*Micropterus salmoides*, LMB). Four diets with varying levels of TPs (0.00%, 0.02%, 0.04%, and 0.08%) were devised to feed LMB with an initial weight of 4.3 ± 0.02 g for 56 days, among which the intermittent feeding (IF) group was fed a diet supplemented with TP8 for 7 days, followed by a basal diet for another 7 days, and this was repeated until the end (56th day). The results demonstrated that supplementation with 0.04% or 0.08% TPs in the diet could reduce the crude lipid content and increase the crude protein content of LMB (*p* < 0.05). The levels of total cholesterol (CHO) and low-density lipoprotein (LDL) in the serum significantly decreased with the addition of 0.08% dietary TPs to the diet (*p* < 0.05). Dietary TPs can stimulate the activities of antioxidant enzymes such as superoxide dismutase (SOD) and catalase (CAT) and enhance the antioxidant capacity of LMB (*p* < 0.05). The activities of immune enzymes such as acid phosphatase (ACP) were increased to improve the immune response via the addition of TPs (*p* < 0.05). Supplementation with 0.02% and 0.04% TPs reduced liver fatty infiltration and alleviated hepatocyte damage. Compared with the control diet, dietary TPs significantly increased villus height (VH), villus width (VW), and lipase (LPS) activity in the intestine (*p* < 0.05), and supplementation with 0.04% TPs significantly increased muscular layer thickness (MT) (*p* < 0.05). With the increase in dietary TPs, distinct differences were observed in the intestinal microbial composition and the relative abundance of potential pathogens, especially *Clostridiaceae*, which decreased, along with the enrichment of pathways related to metabolism, including amino acid metabolism, carbohydrate metabolism, and lipid metabolism. Additionally, intermittent feeding could alleviate the adverse effects caused by a high dosage. In conclusion, dietary TPs of LMB could enhance antioxidant capacity and immunity and improve intestinal health, and intermittent feeding could mitigate the adverse effects caused by a high dosage.

## Simple Summary

This study investigated the effects of tea polyphenols (TPs) on largemouth bass’s growth, antioxidant, and immunity status as well as the intestinal health of the juvenile fish. During a 56-day period, fish were fed diets with varying TP levels. The results found that TPs enhanced the antioxidant capacity and modulated the expression of related immune genes, thereby strengthening immunity; however, high-dose supplementation reduced the antioxidant capacity. In addition, TPs increased the richness and diversity of intestinal microbial communities and decreased the relative abundance of potential pathogens. Overall, adding TPs positively impacted the health and growth of largemouth bass.

## 1. Introduction

China’s aquaculture production accounts for more than half of the global aquatic food production, which is suggested to grow continuously until 2030 [1,2]. Largemouth bass (*Micropterus salmoides*, LMB) has been extensively cultured in China because of its rapid growth, high adaptability, and short reproductive cycle [3], and total production reached 802,400 tons in 2022 [4]. Intensive aquaculture can achieve high production but can also pose high environmental and food safety risks due to water quality deterioration and disease outbreaks in the culture species [5,6]. During intensive aquaculture, antibiotics are commonly used to combat frequent diseases or even as growth promoters to increase productivity [7]. Nonetheless, improper antibiotic use leads to adverse consequences, such as antibiotic residues, disruption of microbial communities, transmission of resistance genes, direct toxic effects on animals, and human health hazards [8,9].

Discharging antibiotic-laden wastewater from aquaculture into rivers can further exacerbate environmental degradation by promoting the spread of antibiotic-resistant bacteria [10,11]. Additionally, the feeding of commercial feeds can lead to changes in patterns of lipid deposition in fish, which can affect their health [12,13]. Therefore, there is an increasing demand for environmentally secure and sustainable strategies in aquaculture.

Phytochemicals such as phenolic compounds, alkaloids, and steroids have been investigated as antibiotic substitutes to promote growth, mitigate oxidative stress, increase antioxidant activity, eliminate pathogens, and improve immune function and disease resistance in major agricultural animals [5,14,15].

Tea polyphenols (TPs), particularly catechins extracted from teas, include four types, namely (−)-epigallocatechin gallate (EGCG), (−)-epigallocatechin (EGC), (−)-epicatechin gallate (ECG), and (−)-epicatechin (EC) [16,17]. TPs have been found to possess antioxidant, immune-enhancing, lipid-modulating, antimicrobial, and antiviral properties [6,16,18,19]. Dietary TPs have been shown to increase superoxide dismutase activity, decrease malondialdehyde content [20], and enhance the resistance to *Aeromonas hydrophila* [21]. In addition, TPs can regulate lipid metabolism-related genes [19] and mitigate fat deposition induced by a high-fat diet [16,22]. Moreover, dietary TPs have been shown to increase feed conversion efficiency and specific growth rates [6,23], while incorporating 4% tea into the diet of *Ictalurus punctatus* markedly diminishes growth metrics, including the rate of weight increase [21]. Nevertheless, research indicates that the overconsumption of TPs could act as an antinutritional factor, potentially suppressing the activities of α-amylase, pepsin, trypsin, and lipase [24]. Furthermore, antinutritional factors can adversely impact the gut microbiome, potentially resulting in intestinal permeability that enables harmful bacteria to enter the lamina propria [25]. Fortunately, alternate use or intermittent feeding can eliminate these negative effects [26].

Therefore, this study aimed to evaluate the effects of TP supplementation on the growth, antioxidant capacity, immune function, and intestinal health of largemouth bass. Moreover, the possible regulatory mechanism was explored by examining the intestinal microbiota alongside the mRNA expression levels of genes associated with antioxidant and immune responses. The results of this research are expected to yield significant insights into the use of TPs as additives in fish feed, offering innovative alternatives to antibiotics and promoting sustainable practices in aquaculture.

## 2. Materials and Methods

### 2.1. Feed Design and Preparation

Tea polyphenols (97%, CAS:84650-60-2) were purchased from Shanghai Macklin Biochemical Technology Co., Ltd. (Shanghai, China). Four isonitrogenous and isolipidic diets were designed with TP levels of 0.00%, 0.02%, 0.04%, and 0.08% and were named as C, TP2, TP4, and TP8, respectively (Table 1). The intermittent feeding (IF) group was fed a diet supplemented with TP8 for 7 days, followed by a basal diet for another 7 days, and this was repeated until the end (56th day). The ingredients for the diets were meticulously ground to a fine powder, accurately measured, and thoroughly mixed with water and oil. Following this process, particles measuring one millimeter in diameter were generated using a twin-screw extruder and a crusher, after which they were air-dried and stored for preservation. All feed constituents and apparatus utilized were supplied by Zhuhai Huolibao Feed Co., Ltd. (Zhuhai, China).

### 2.2. Experimental Trial

All procedures involving the fish were carried out in line with the “Guiding Principles in the Care and Use of Animals (China)” and received approval from the Animal Welfare Division of South China Agricultural University in Guangzhou, China (2023G024). The juvenile LMB used in this study was acquired from Guangdong Weilai Biotechnology Co., Ltd. in Guangzhou, China. Prior to the trial, the fish were acclimatized and provided with a 0.00% TPs diet twice daily for a period of seven days within a recirculating aquaculture system. Following this, 450 healthy fish of comparable size (average weight of 4.3 ± 0.02 g) were randomly distributed into fifteen glass tanks (each holding 150 L, with three tanks allocated per group), containing thirty fish per tank. All the fish were fed twice (at 9:00 a.m. and 4:00 p.m. daily) to satiation, and the daily feed intake was recorded. All glass tanks were maintained under a natural light regime (from 1 October to 25 November), and the culture water was continuously aerated in a closed recirculating aquaculture system with dissolved oxygen maintained above 7.2 mg/L, a temperature of 29.2 ± 1.2 °C, a pH of 6.95–7.65, and ammonia nitrogen and nitrite concentrations below 0.1 mg/L.

### 2.3. Sampling

Following the 56-day feeding trial, the fish were deprived of food for a period of 24 h. Before taking samples, the quantity and weights of the fish in each tank were documented, and six fish from each tank were randomly chosen to measure their length and weight. Six fish were randomly selected from each tank and promptly anesthetized (eugenol 50 mg/L); blood samples were obtained from the tail vein, and serum samples were prepared according to the methods described by Liao et al. [27] and stored at −80 °C for testing physiological and biochemical indicators. Following the collection of blood, samples from the liver, midgut, and intestinal contents were swiftly frozen in liquid nitrogen and subsequently preserved at −80 °C for the assessment of enzyme activity markers, real-time PCR analysis, and the evaluation of intestinal microbial components. Additionally, two more fish per tank were anesthetized, and liver and midgut samples were collected and preserved in 4% paraformaldehyde for histological examination. The dorsal muscle was collected, and two additional fish per tank were preserved at −20 °C for proximate composition analysis. For all the control and experimental groups, three biological replicates were analyzed, with each biological replicate being a mixture of six fish for serum and dorsal muscle, three fish for liver and intestinal tissues, and two fish for whole body proximate composition in equal proportions.

### 2.4. Growth Parameters

The growth parameters of the experimental fish were calculated according to the following formulae:

Survival rate (SR, %) = (final number/initial number) × 100;

Weight gain rate (WGR, %) = (final body weight − initial body weight)/initial body weight) × 100;

Specific growth rate (SGR, %/day) = 100 × [ln (final body weight) − ln (initial body weight)]/days;

Feed conversion ratio (FCR) = dry feed intake/(final body weight − initial body weight);

Food intake (FI, %/day) = dry feed consumed × 100 × 2/[days × (initial body weight + final body weight + dead fish body weight)];

Condition factor (CF, g/cm^3^) = (final body weight/body length^3^) × 100;

Viscerosomatic index (VSI, %) = (visceral mass weight/body weight) × 100;

Hepatosomatic index (HSI, %) = (liver weight/body weight) × 100.

### 2.5. Proximate Composition Assay

Determinations of moisture and proximate compositions (crude protein, crude lipids, and crude ash) in the diets, whole fish, and muscles were referred to the description by Liao et al. [27] and Zhang et al. [28].

### 2.6. Enzymatic Activities and Serum Biochemical Indices

Liver and intestinal samples were blended in a 1:9 (*w*/*v*) saline solution at ice-water temperature, followed by centrifugal separation to collect the supernatant. The measurements of superoxide dismutase (SOD) (A001–3-2), catalase (CAT) (A007-1-1), malondialdehyde (MDA) (A003–1-2), total antioxidant capacity (T-AOC) (A015–2-1), acid phosphatase (ACP) (A060-2), lipase (LPS) (A054-2-1), and amylase (AMS) (C016-1-1) were conducted using diagnostic reagent kits following the provided guidelines from the Nanjing Jiancheng Bioengineering Institute in China. Results were analyzed with a Synergy HTX multimode reader manufactured by BioTek Instruments, Inc., Winooski, VT, USA. The procedures were outlined in the study by Zhang et al. [28].

Serum alanine aminotransferase (ALT), aspartate aminotransferase (AST), alkaline phosphatase (ALP), trilaurin (TG), total cholesterol (CHO), high-density lipoprotein (HDL), and low-density lipoprotein (LDL) levels were determined by a commercial company (Wuhan Service Biotechnology, Wuhan, China) via standard methods with an automatic biochemical analyzer (Rayto Life and Analytical Sciences Co., Ltd., Shenzhen, China).

### 2.7. Histology Analysis

The liver and intestinal samples were dispatched to Wuhan Servicebio Technology Corporation in Wuhan, China, where they underwent paraffin embedding and received hematoxylin and eosin (H&E) staining. Following this, the histological micrographs were carefully examined, and key characteristics were documented using a Nikon Eclipse Ci-L light microscope equipped with an advanced imaging system.

### 2.8. Real-Time PCR Assay

RNA from the liver was isolated using a total RNA extraction kit (model 9767, Takara, Tokyo, Japan). cDNA synthesis was achieved with the PrimeScript TMRT reagent kit (product code RR0047A, Takara, Tokyo, Japan). Refer to Table 2 for the primers sequences utilized in the current research. The PCR reactions were carried out using the SYBR Green Master Mix (batch QPS-201T, Toyobo, Osaka, Japan) within the CFX Connect Real-Time System (manufactured by Bio-Rad Laboratories, Hercules, CA, USA). The relative expression levels of all the target genes were quantified via the 2^−ΔΔCT^ method.

### 2.9. Intestinal Microbiota Analysis

The icy gastrointestinal material from the fish in the C, TP4, and TP8 groups was then shipped off to Majorbio Biopharm Technology Co., Ltd. in Shanghai, China, for a deep dive into their intestinal flora using 16S next-generation sequencing. In brief, we pulled out the DNA and ran PCR with the usual 338 F and 806 R primers, resulting in amplicons that were roughly 450 base pairs in length. The PCR products were first quantified, homogenized, and then randomly chosen for Illumina MiSeq library construction and sequencing with the Illumina MiSeq PE300 platform. Before bioinformatics analysis, the raw data were subjected to quality control via the Fastp software (version 0.19.6), and sequence splicing was subsequently performed via the Flash software (version 1.2.11). The sequences were clustered at 97% similarity via the Uparse software (version 11) for OTUs. Species classification of the sequences was achieved using the RDP Classifier (version 2.13), while OTU assignments were made through the SILVA database (release 138). Alpha diversity assessments were conducted using the Mothur software (version 1.30.2); beta diversity was assessed via principal coordinate analysis (PCoA), and taxonomic level abundance was determined with QIIME (version 1.9.1). KEGG and COG functional prediction analyses were performed with PICRUSt2 (version 2.2.0), and the Kruskal–Wallis H test was employed to validate the KEGG functional variances.

### 2.10. Statistical Analysis

Experimental data were analyzed with the SPSS software (version 25.0) using a one-way ANOVA, followed by multiple comparison analyses employing Duncan’s test. Statistical significance was deemed to exist with a *p*-value less than 0.05. The results are displayed as the mean of the treatment ± standard error of the mean (Mean ± SEM, *n* = 3). The figures were generated and enhanced using GraphPad Prism 6 and Adobe Photoshop CC 2019.

## 3. Results

### 3.1. Growth Performance, Proximate Composition, and Biometric Parameters

As shown in Table 3, there were no significant differences in growth indices, including SR, FBW, WGR, SGR, FCR, and FI (*p* > 0.05), although the average FBW and WGR were much lower in the TP4 and TP8 groups than in the other three groups. The FCR ranged from 0.79 to 0.81 in all the groups. The CF values for the TP2, TP4, and TP8 groups were significantly greater than those for the C group (*p* < 0.05), whereas the VSI and HSI values for the TP8 group were significantly lower than those for the other groups (*p* < 0.05). Compared with those of the control and TP2 groups, the crude lipid contents of the whole body and muscle of the TP4, TP8, and IF groups were significantly lower (*p* < 0.05). The muscle crude protein content in the TP2 and IF groups was significantly greater than in the control groups (*p* < 0.05).

### 3.2. Serum Biochemical Indices

The serum biochemical indices are shown in Table 4. The levels of CHO and LDL in the serum tended to decrease as the dietary content of the TPs increased (*p* < 0.05). Compared with the control group, the TP2 group presented a significantly greater level of ACP (*p* < 0.05). However, there were no significant differences in the other indices among the different groups (*p* > 0.05).

### 3.3. Antioxidant Capacity of the Serum and Liver

**Figure 1:**
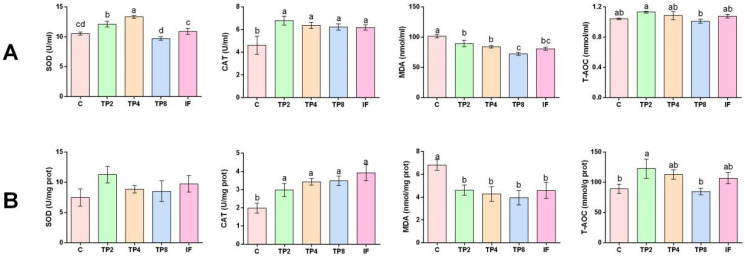
Serum and liver antioxidant enzyme activities of largemouth bass fed the five experimental diets. (**A**) Enzyme activity of the serum; (**B**) enzyme activity of the liver. SOD (superoxide dismutase); CAT (catalase); MDA (malondialdehyde); T-AOC (total antioxidant capacity). Different letters indicate significant differences (*p* < 0.05), and the same letter or no letter indicates no significant difference (*p* > 0.05).

The serum SOD activity tended to increase, reached its highest level in the TP4 group, and then decreased as the TP level increased further (*p* < 0.05) (Figure 1A). Compared with the control group, the TP2, TP4, TP8, and IF groups presented significantly greater serum CAT activity (*p* < 0.05) (Figure 1A). The serum MDA content tended to decrease as the TP concentration increased, with the lowest content in the TP8 group (*p* < 0.05) (Figure 1A). The T-AOC level in the TP2 group was significantly greater than in the TP8 group, despite the absence of notable differences among the other groups (*p* > 0.05) (Figure 1A).

The activity of liver SOD did not significantly differ among the groups (Figure 1B). The activity of CAT in the TP2, TP4, TP8, and IF groups was significantly greater than in the control group (*p* < 0.05) (Figure 1B). The MDA content in the four experimental groups was significantly lower than in the control group, with the lowest value occurring in the TP8 group (*p* < 0.05) (Figure 1B). The T-AOC level in the TP2 group was significantly greater than in the C and TP8 groups (*p* < 0.05) (Figure 1B). Moreover, the SOD activity levels in TP2, TP4, TP8, and IF were on average higher than in the remaining groups, albeit the difference was not statistically notable (*p* > 0.05) (Figure 1B).

### 3.4. Digestive Enzyme Activities

**Figure 2:**
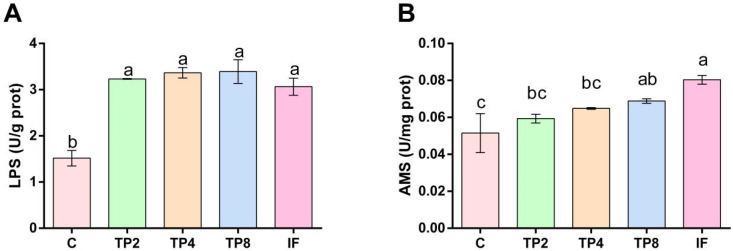
Intestinal digestive enzyme activities of largemouth bass fed the five experimental diets. (**A**) LPS (lipase); (**B**) AMS (amylase). Different letters indicate significant differences (*p* < 0.05), and the same letter indicates no significant difference (*p* > 0.05).

The midgut LPS activity was two times greater in the four experimental groups than in the control group (*p* < 0.05) (Figure 2A). AMS activity generally rose with higher dietary TP levels, showing significant increases in the TP8 and IF groups compared with the C group (*p* < 0.05) (Figure 2B).

### 3.5. Intestine and Liver Histomorphology

**Figure 3:**
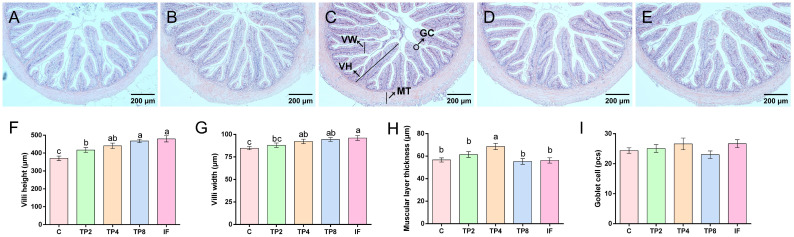
Intestinal tissue morphology of largemouth bass fed the five experimental diets. (**A**–**E**) C, TP2, TP4, TP8, and IF (100X), respectively. VH (villi height); VW (villi width); MT (muscular layer thickness); GC (goblet cell). (**F**–**I**) Villus height, villus width, muscular layer thickness and goblet cell number, respectively. Different letters indicate significant differences (*p* < 0.05), and the same letter or no letter indicates no significant differences (*p* > 0.05).

Figure 3 presents the intestinal segments and their corresponding histological findings. Compared with those in the control group, the fish in the TP2, TP4, TP8, and IF groups presented significantly greater intestinal villus heights (*p* < 0.05). Furthermore, the intestinal villus width in the TP4, TP8, and IF cohorts was notably wider compared to the C group (*p* < 0.05). The intestinal muscular layer’s thickness in the TP4 cohort was notably more extensive compared to other groups (*p* < 0.05). However, there were no significant differences in the number of goblet cells among the groups (*p* > 0.05).

**Figure 4:**
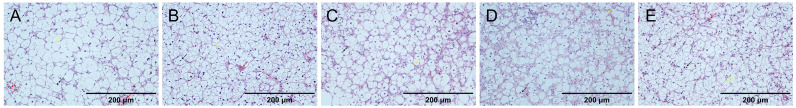
Hematoxylin–eosin-stained liver sections from largemouth bass fed the five experimental diets. Figures (**A**–**E**) represent groups C, TP2, TP4, TP8, and IF (200×), respectively. The red arrows represent congestion; the black arrows represent off-center hepatocyte nuclei, and the yellow arrows represent lipid vacuoles.

The liver section findings are shown in Figure 4. The liver of largemouth bass exhibited apparent fatty infiltration, indicating varying degrees of damage. In the control group, hepatocyte nuclei were expelled towards the cell periphery, accompanied by a heightened and pronounced loss of nuclei and signs of congestion (Figure 4A). However, the livers in the TP2 and TP4 groups displayed clearer cell membrane demarcation, with reduced fatty infiltration and significantly improved hepatocyte breakdown (Figure 4B,C).

### 3.6. Intestinal Microbial Analysis in the C, TP4, and TP8 Groups

#### 3.6.1. Diversity, Richness, and Structure of the Intestinal Microbiota

**Figure 5:**
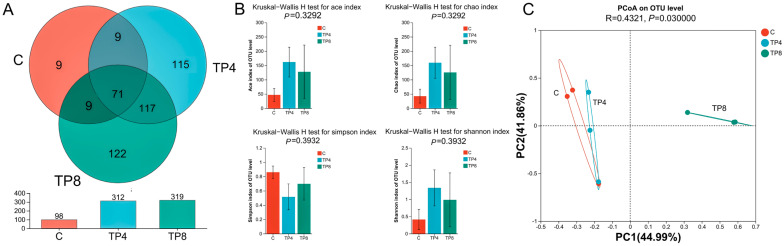
Venn diagram, alpha diversity, and beta diversity of the intestinal microbiota. (**A**) Venn diagram; (**B**) alpha diversity analysis, Ace index, Chao index, Simpson index, and Shannon index; (**C**) PCoA based on the Bray–Curtis distance algorithm.

According to the Venn diagram, the TP8 group had the greatest number of intestinal microbial OTUs, whereas the control group had the lowest number of OTUs (Figure 5A). The C, TP4, and TP8 groups contained 9, 115, and 122 unique OTUs, respectively. Additionally, the three groups shared a total of 71 OTUs. The TP4 group exhibited the highest averages for the Shannon, Chao, and Ace indices, yet the disparities were not statistically significant (*p* > 0.05) (Figure 5B).

Figure 5C illustrates the PCoA outcomes, with the PC1 and PC2 explaining 44.99% and 41.85%, respectively (R = 0.4321, *p* = 0.030000). The samples in the TP4 and C groups overlapped, indicating that their microbial compositions were relatively similar. The samples in the TP8 group were located in different quadrants with significant deviations from those in the TP4 and C groups, indicating that their microbial compositions were significantly different.

#### 3.6.2. Bacterial Composition

**Figure 6:**
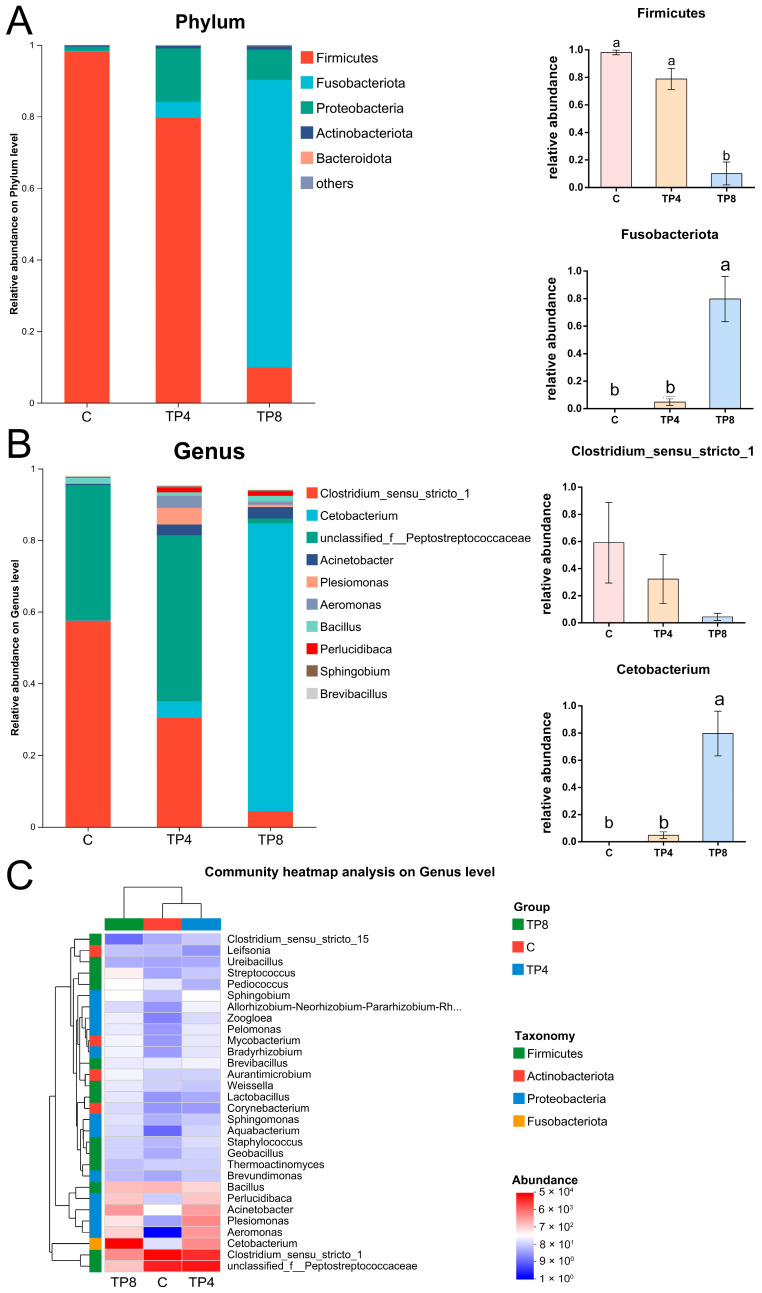
Composition of the intestinal microbiota. (**A**) Relative abundance of species at the phylum level; (**B**) relative abundance of species at the genus level (top 10); (**C**) clustering heatmap analysis of the intestinal microbiota at the genus level. Abundances less than 0.1% were classified as others. Different letters indicate significant differences (*p* < 0.05), and the same letter or no letter indicates no significant difference (*p* > 0.05).

At the phylum level, Firmicutes was the most abundant phylum in both the C and TP4 groups (98.24%, 79.60%), which was significantly greater than in the TP8 group (9.79%) (*p* < 0.05), whereas Fusobacteriota was the most abundant phylum in the TP8 group (80.43%) (*p* < 0.05) (Figure 6A). In terms of genus, the dominant species within the gut microbiome were primarily *Clostridium_sensu_stricto_1*, *Cetobacterium,* and *unclassified_f_Peptostreptococcaceae*. Notably, the relative abundance of *Cetobacterium* was notably greater in the TP8 group (80.43%) compared to the C and TP4 groups (0.18%, 4.48%) (*p* < 0.05). Although the relative abundance of *unclassified_f_Peptostreptococcaceae* was lower (9.79%), the differences were not statistically significant (*p* > 0.05) (Figure 6B).

According to the genus-level heatmap (Figure 6C), the C and TP4 groups presented similar species compositions. Compared with those in the TP4 group, the relative abundances of *Streptococcus*, *Pediococcus*, *Aurantimicrobium*, *Weissella*, *Lactobacillus*, *Corynebacterium*, *Bacillus*, *Acinetobacter*, and *Cetobacterium* increased, whereas the relative abundances of *Clostridium_sensu_stricto_1*, *Perlucidibaca*, *Plesiomonas*, *Aeromonas*, *Clostridium_sensu_stricto_1*, and *unclassified_f__Peptostreptococcaceae* decreased in the TP8 group. However, compared with those in the TP8 group, the relative abundances of *Clostridium_sensu_stricto_1* and *unclassified_f__Peptostreptococcaceae* significantly increased, whereas the relative abundances of *Streptococcus*, *Pediococcus*, *Sphingobium*, *Perlucidibaca*, *Acinetobacter*, *Plesiomonas*, *Aeromonas*, and *Cetobacterium* decreased in the C group.

**Figure 7:**
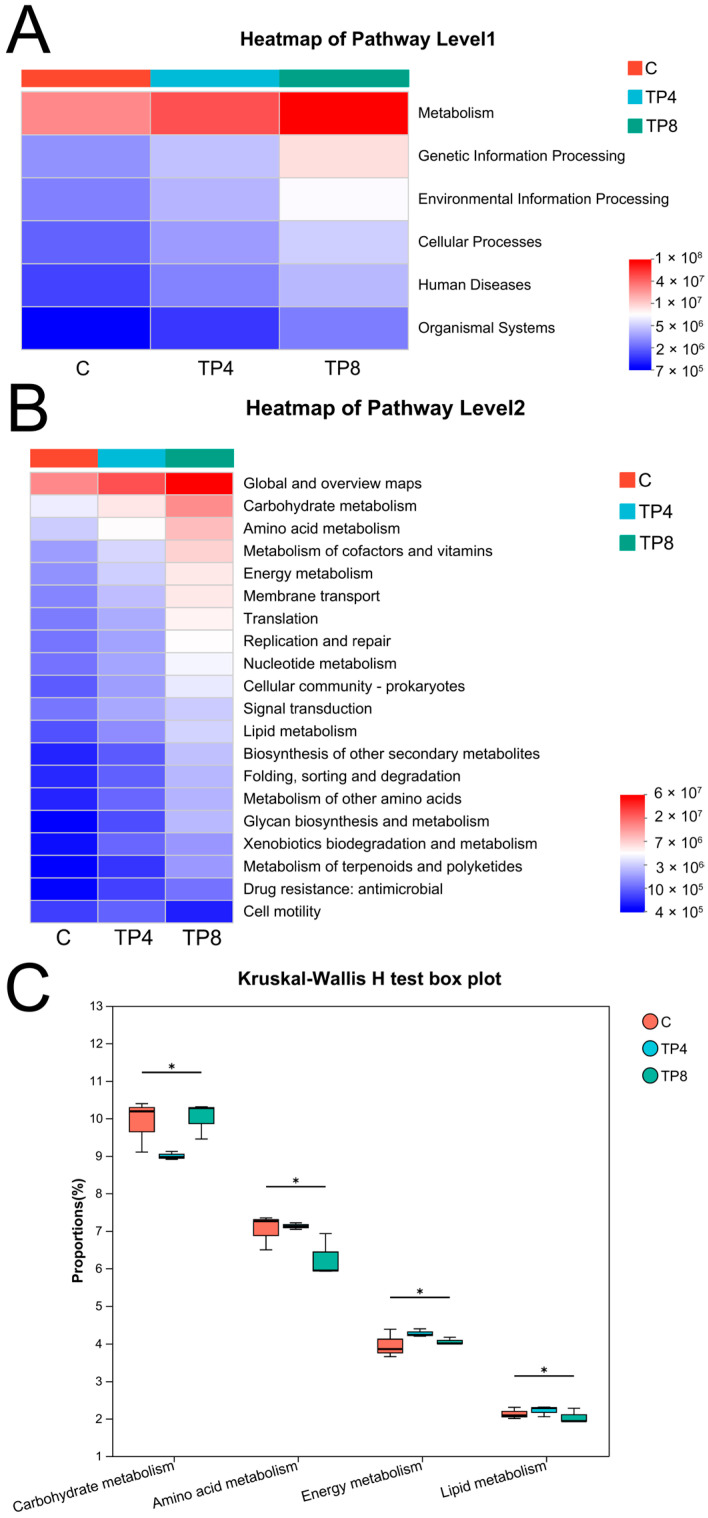
Functional predictive analysis of the intestinal microbiome. (**A**) Functional pathway level 1 based on KEGG; (**B**) The top 20 functional pathway level 2 based on KEGG; (**C**) KEGG functional difference analysis based on the Kruskal–Wallis H test. * *p* < 0.05.

KEGG metabolic pathway analysis revealed that the gut microorganisms of the largemouth bass were primarily involved in metabolism, followed by genetic information processing and environmental information processing (Figure 7A). The results of the Kruskal–Wallis H test revealed that 18 of the top 20 KEGG functional pathways were significantly different between the C and TP8 groups (*p* < 0.05) (Figure 7B and Figure S1). Specifically, significant variations were detected in metabolic pathways, including carbohydrate metabolism, amino acid metabolism, energy metabolism, and lipid metabolism (*p* < 0.05) (Figure 7C).

### 3.7. The mRNA Expression of Genes Related to Antioxidation and Immunity

**Figure 8:**
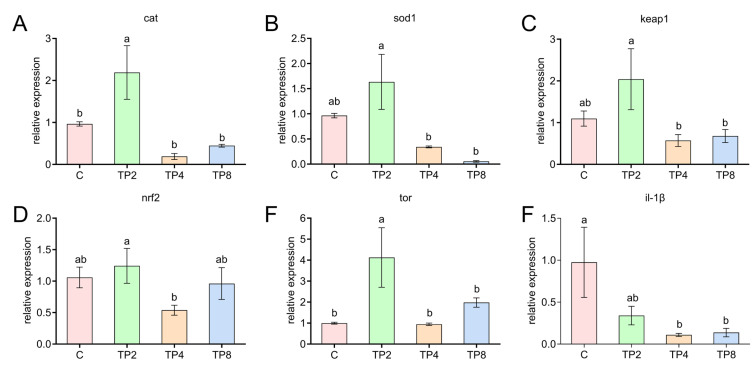
Expression levels of genes related to antioxidation and immunity in the liver. (**A**) CAT (catalase); (**B**) SOD1 (superoxide dismutase 1); (**C**) Keap1 (kelch-like ech-associated protein 1); (**D**) Nrf2 (nf-e2-related factor 2); (**E**) TOR (target of rapamycin); (**F**) IL-1β (interleukin 1β). Different letters indicate significant differences (*p* < 0.05), and the same letter indicates no significant differences (*p* > 0.05).

The mRNA levels of antioxidant- and immune-related genes in the C, TP2, TP4, and TP8 groups are shown in Figure 8. In the liver, the mRNA levels of Keap1, Nrf2, CAT, SOD1, and TOR in the TP2 group were significantly greater than those in the other groups (*p* < 0.05). However, the mRNA levels of IL-1β in the TP4 and TP8 groups were significantly lower than those in the control group (*p* < 0.05).

## 4. Discussion

In this study, the WGR of the largemouth bass was highest when 0.02% TPs were added and decreased with increasing concentration, but there were no significant differences among all the groups. Similar results were also reported in large yellow croaker when 0.01~0.05% TPs was added to the diet [19]. However, when fed a diet supplemented with 3% broken green tea, the growth of yellow river carp significantly increased [22], and dietary TPs addition at 0.05% improved the growth performance of grass carp at various protein and carbohydrate ratios [34]. These results suggest that incorporating TPs into fish feed does not uniformly lead to similar growth benefits. The discrepancies observed across these studies may stem from differences in dietary compositions, the amounts or types of TPs used, and the various fish species involved [6].

In the present research, the trend toward decreased growth performance in the TP4, TP8, and IF groups was probably due to decreased lipid deposition as the whole-body and muscle crude lipid contents in these groups significantly decreased. Furthermore, decreased HSI and VSI along with lower levels of CHO and LDL in the serum support the idea that TPs may contribute to the reduction in both body and blood lipid levels, as was also proven by research on yellow croaker and other mammals, such as humans [19,35].

Artificially formulated diets caused apparent fatty infiltration and injury to the liver in largemouth bass [36], as was also shown in the present study. Compared with the control, liver histology in the TP2 and TP4 groups revealed reduced fatty infiltration and significantly improved hepatocyte breakdown, suggesting that TPs have hepatoprotective effects, and these finding are similar to those of previous studies [37,38]. However, the improvement in the TP8 group was not as good as that in the low-dose groups, which suggested that a high-dose intake of TPs has hepatotoxic effects, as TPs or EGGC can lead to severe liver injury or disorders in humans [39]. However, liver damage in fish has not been reported. Follow-up experiments are needed to determine the optimal addition dose.

Intestinal histomorphology and digestive enzyme activities are important indicators of digestion and absorption capacity [30]. Increased intestinal MT, VH, and VW suggest healthier intestines and better digestion ability in fish [40,41]. In the present research, combined with the above histomorphology indicators, the intestinal structure of the TP4 group was the healthiest. Previous research revealed that 0.012% TPs enhanced grass carp health [42], and 0.03% TPs resulted in the best intestinal morphology of juvenile hybrid sturgeon (*Acipenser baerii* ♀ × *A. schrenckii* ♂) [40]. Notably, intestinal LPS activity increased, which is consistent with the previous finding that adding a moderate amount of TPs significantly enhances intestinal LPS activity [6,23].

TPs are widely reported to have antioxidative effects on animals and humans. When fish are exposed to environmental stress or assault by bacteria or viruses, reactive oxygen species (ROS) generation intensifies, and excessive ROS can lead to oxidative stress [43]. Antioxidant enzymes such as SOD and CAT play a crucial role in protecting cells and tissues from oxidative stress, with their activity reflecting health status and metabolic balance of fish [44]. The T-AOC represents the total antioxidant level, which consists of various antioxidant enzymes and substances and reflects the overall antioxidant capacity of a biological system [43,45]. However, MDA is a lipid peroxidation product and is detrimental to cells and tissues, serving as an indicator of the degree of damage caused by oxygen free radicals [46]. Ma et al. [47] applied TPs to a culture of grass carp and conducted a virus challenge test. The authors reported that the addition of TPs increased SOD and CAT activities and decreased the MDA content in the gills, and they concluded that TPs can improve gill oxidative status and maintain healthy conditions. They also reported that high doses (≥0.02%) of TPs significantly reduced SOD and CAT activities, which was attributed to the metal-chelating abilities of TPs. TPs may inhibit the antioxidant system if added excessively [48]. Similarly, the results of this research demonstrated that the SOD activity in the serum and the CAT activity in the liver and serum increased, and that the MDA content decreased in the TPs addition group, but high-dose supplementation reduced the SOD activity. Nevertheless, the SOD activity in the IF group was greater than in the TP8 group, suggesting that intermittent feeding could alleviate the adverse effects evoked by a high dosage. Similarly, the serum and liver T-AOCs in the TP2 group were greater than those in the other groups, and when the TPs increased further, the T-AOC decreased gradually, suggesting that low-level TPs can promote the scavenging of free radicals and enhance the antioxidant response of largemouth bass; however, high-dose supplementation reduced the antioxidant capacity.

Fish nonspecific immunity, also known as innate immunity, consists of antibacterial, antiviral, and antiparasitic responses, with the antibacterial response being the dominant response [43]. ACP and ALP can increase pathogen recognition and phagocytosis and increase disease resistance in fish [49]. The current research revealed that the serum ACP activity in the TP2 group was notably higher than in the control group, suggesting that TPs play a beneficial role in boosting the immune response of largemouth bass.

On the basis of enzyme activity, the changes in the transcription of antioxidant-related genes in the control, TP2, TP4, and TP8 groups were further detected. The Nrf2–Keap1 signaling pathway is a crucial mechanism for alleviating oxidative stress and is activated by reducing the levels of Keap1 and increasing the activity and gene expression of Nrf2 [50,51]. TPs have been shown to lower the levels of Keap1 and increase the expression of Nrf2-targeted genes [47,52]. However, in the TP2 group, there was a notable elevation in the mRNA expressions of keap1, nrf2, cat, and sod1 within the livers. This inconsistency may be attributed to the fact that TPs can regulate Nrf2 expression via a Keap1-independent mechanism.

Oxidative stress induces cellular immunosuppression, and the immune response in fish is closely associated with inflammatory cytokines [47,53]. Cytokines, including interleukins (ILs), play crucial roles in host innate immunity [54]. IL-1β is considered an essential proinflammatory cytokine [29]. The expression of IL-1β in the liver decreased, indicating that TPs may reduce the level of proinflammatory factors to resist the inflammatory response. This finding aligns with the findings of Nootash et al. [55], who supplemented rainbow trout diets with green tea. TOR is a ubiquitous protein kinase whose dysregulation has been implicated in numerous metabolic diseases [56,57]. Previous studies have indicated that L-theanine can increase mTOR gene expression, which initially increases but then decreases as the level of L-theanine increases, suggesting that L-theanine improves the immune function of female Chinese mitten crabs [58]. Similar findings were reported in this study, suggesting that TPs may also enhance immune function. When pieced together, the findings suggest that incorporating dietary TPs can bolster the antioxidant and immune defenses in the liver of largemouth bass. However, the specifics of this research are not detailed enough, and a more comprehensive study is crucial to unravel the precise working of these TPs.

The intestinal microbiota colonizes the host intestine and plays crucial roles in maintaining homeostasis, digestion, metabolism, and immune functions [59,60]. Its richness constitutes an essential indicator of host health, and greater diversity is correlated with protection from foreign microbes [18]. In the present study, the TP4 and TP8 groups presented greater numbers of OTUs than the C group, but there were no significant differences in α diversity. Moreover, PCoA demonstrated that the C and TP4 groups exhibited a good clustering tendency, suggesting that high-level TPs changed the composition of the gut microbiota of largemouth bass, which is similar to the findings of Loach [61]. These findings suggest that TPs enhance microbial community stability through elevated richness and diversity.

At the phylum level, the prevalent bacterial groups across the three samples were Firmicutes, Fusobacteria, and Proteobacteria, constituting the primary intestinal bacteria of largemouth bass [62,63]. *Cetobacterium*, the predominant species within in the Fusobacteria, is a vitamin B12 (B12)-producing bacteria [64], and B12 deficiency dysregulates lipid metabolism, leading to lipid accumulation [65]. The significant increase in the abundance of *Cetobacterium* (from 0.18% to 80.43%) in the TP8 group was probably responsible for the significant decrease in crude lipids. Firmicutes and Bacteroidetes can metabolize various complex carbohydrates [66], and their ratios are related to the development of obesity [67]. The gut microflora of obese animals and humans has increased F/B ratios [68]. Previous studies have shown that TPs exert an inhibitory effect on Firmicutes and Bacteroides [69,70], and that tea extract can significantly reduce the F/B ratio in obese mice [71]. In this study, TP treatments led to a significant decrease in the abundance of Firmicutes and *Clostridium* and a slight decrease in the abundance of Bacteroidetes, resulting in a decreased F/B ratio, which is consistent with previous findings [39,70,72]. The prediction of gut microbial functions verified that TPs regulate metabolism-related pathways and exhibit antiobesity biological activities [73,74]. In this study, TPs modulated pathways associated with carbohydrate metabolism, amino acid metabolism, energy metabolism, and lipid metabolism, indicating that TPs can reduce lipid content, and that appropriate supplementation with TPs could assist the microbiota in efficiently utilizing nutrients. The above results demonstrate that TPs can improve the intestinal microbial composition and enhance metabolism and intestinal barrier integrity. Dietary TPs positively influence the intestinal health of largemouth bass. However, further research is required to ascertain if the overuse of TPs could provoke enteritis.

## 5. Conclusions

The supplementation of dietary TPs can reduce lipid accumulation and increase the crude protein content of LMB. Additionally, dietary TPs can enhance the function of antioxidant and immune enzymes, boosting the antioxidant capacity and immune response of LMB. Furthermore, the analyses of digestive enzyme activities, intestinal histomorphology and the intestinal microbiota indicate that supplementation with TPs can maintain intestinal microbiota homeostasis and improve the intestinal health of LMB. Additionally, intermittent feeding can mitigate the adverse impacts caused by a high dosage. In summary, the present study suggests that TPs can be employed as a feed additive for LMB. Nevertheless, additional studies are required to identify the ideal dosage schedules and elucidate the fundamental mechanisms to maximize the advantages of TPs in aquaculture practices.

## Figures and Tables

**Table 1:** Formulation and composition of experimental diets (% of dry matter basis).

Ingredients (%)	C	TP2	TP4	TP8
Fish meal	45	45	45	45
Fermented soybean meal	14	14	14	14
Corn gluten meal	10	10	10	10
Chicken meal	5	5	5	5
Fish oil	3	3	3	3
Soybean oil	3	3	3	3
High-gluten flour	15	15	15	15
Ca(H_2_PO_4_)_2_	1	1	1	1
Choline chloride	0.2	0.2	0.2	0.2
Tea polyphenols	0	0.02	0.04	0.08
Bentonite	1.8	1.78	1.76	1.72
Vitamin mineral mixture ^1^	2	2	2	2
Total	100	100	100	100
Proximate composition				
Moisture (%)	6.67	7.34	5.76	7.21
Crude protein (%)	47.79	47.91	48.36	48.67
Crude lipid (%)	6.07	7.05	6.33	6.17
Crude ash (%)	12.69	12.45	13.39	12.05

Note: Composition data are the means of three replicate measurements. ^1^ Vitamins (per kg diet): vitamin A: ≥3,500,000 IU; vitamin D3: 1,000,000 IU; vitamin E: ≥40 g; vitamin K3: ≥4 g; vitamin B1: ≥8 g; vitamin B2: ≥8 g; vitamin B6: ≥8 g; vitamin B12: ≥15 mg; vitamin C: ≥150 g; D biotin: ≥80 mg; nicotinamide: ≥30 g; D-Calcium pantothenate: ≥15 g; folic acid: ≥2.5 g; and inositol: ≥80 g. Minerals (per kg diet): K ≥ 42 g; Mg: ≥8.3 g; Fe: 15–30 g; Zn: 3.5–7 g; Mn: 2.3–4.6 g; Cu: 0.81–1.62 g; Co: 0.21–0.42 g; I: 0.11–0.22 g; and Se: 0.02–0.04 g. Vitamins and minerals were provided by Guangzhou Lijia Biotechnology Co., Ltd. (Guangzhou, China).

**Table 2:** Primers used for real-time PCR gene expression assays.

Gene	Forward (5-3′)	Reverse (5-3′)	Source
Keap1	GCACCTAACCGTGGAACTCAA	CCAGTTTTAGCCAGTCATTGTTCC	[29]
Nrf2	TCACCAAAGACAAGCGTAA	CAGGCAGATTGATAATCATAGA	[30]
CAT	GTTCCCGTCCTTCATCCACT	CAGGCTCCAGAAGTCCCACA	[29]
SOD1	CCCCACAACAAGAATCATGC	TCTCAGCCTTCTCGTGGA	[31]
TOR	TCAGGACCTCTTCTCATTGGC	CCTCTCCCACCATGTTTCTCT	[32]
IL-1β	AGCACCCTCGTGTCTGTTG	CAGGTTTCAACTCTGACGCT	[33]
β-actin	ATCGCCGCACTGGTTGTTGAC	CCTGTTGGCTTTGGGGTTC	[32]

Note: Keap1, kelch-like ech-associated protein 1; Nrf2, nf-e2-related factor 2; CAT, catalase; SOD1, superoxide dismutase 1; TOR, target of rapamycin; IL-1β, interleukin 1β.

**Table 3:** Growth performance, biometric parameters, and proximate composition of largemouth bass fed the five experimental diets.

Growth Performance	C	TP2	TP4	TP8	IF
SR (%)	96.67 ± 1.92	95.56 ± 2.22	100 ± 0.00	96.67 ± 1.92	100 ± 0.00
IBW (g)	4.29 ± 0.05	4.31 ± 0.08	4.29 ± 0.04	4.22 ± 0.01	4.37 ± 0.02
FBW (g)	37.2 ± 0.9	37.9 ± 0.9	34.2 ± 1.6	34.9 ± 1.0	35.5 ± 0.9
WGR (%)	737.0 ± 17.0	741.6 ± 33.4	696.9 ± 31.6	697.9 ± 18.7	712.4 ± 22.6
SGR (%/day)	3.79 ± 0.04	3.8 ± 0.07	3.7 ± 0.07	3.71 ± 0.04	3.74 ± 0.05
FCR	0.8 ± 0.01	0.82 ± 0.02	0.79 ± 0.01	0.8 ± 0.02	0.81 ± 0.03
FI (%/day)	2.18 ± 0.03	2.21 ± 0.03	2.2 ± 0.04	2.16 ± 0.06	2.26 ± 0.08
Biometric parameters					
CF (g/cm^3^)	2.02 ^b^ ± 0.08	2.29 ^a^ ± 0.03	2.27 ^a^ ± 0.02	2.2 ^a^ ± 0.04	2.17 ^a^ ± 0.03
VSI (%)	9.64 ^a^ ± 0.10	9.37 ^a^ ± 0.05	9.55 ^a^ ± 0.34	8.38 ^b^ ± 0.33	9.43 ^a^ ± 0.20
HIS (%)	4.5 ^a^ ± 0.12	3.94 ^a^ ± 0.27	4.33 ^a^ ± 0.48	3.24 ^b^ ± 0.23	4.07 ^a^ ± 0.16
Whole body composition					
Moisture (%)	71.7 ^b^ ± 0.0	72.1 ^ab^ ± 0.5	72.7 ^ab^ ± 0.8	71.9 ^b^ ± 0.6	75.8 ^a^ ± 2.2
Crude protein (%)	17.2 ± 0.5	18.2 ± 0.1	18.0 ± 0.2	18.0 ± 0.1	16.2 ± 1.5
Crude lipid (%)	6.74 ^a^ ± 0.11	6.15 ^ab^ ± 0.55	5.04 ^bc^ ± 0.65	4.41 ^c^ ± 0.57	3.88 ^c^ ± 0.25
Crude ash (%)	3.66 ± 0.07	3.5 ± 0.28	3.8 ± 0.32	3.66 ± 0.08	3.67 ± 0.82
Muscle composition					
Moisture (%)	77.5 ± 0.2	77.2 ± 0.3	77.4 ± 0.2	77.6 ± 0.1	77.3 ± 0.1
Crude protein (%)	19.7 ^b^ ± 0.25	20.3 ^a^ ± 0.03	20.0 ^ab^ ± 0.0	20.1 ^ab^ ± 0.1	20.2 ^a^ ± 0.1
Crude lipid (%)	1.9 ^a^ ± 0.06	1.98 ^a^ ± 0.05	1.36 ^b^ ± 0.21	1.04 ^b^ ± 0.13	1.24 ^b^ ± 0.06
Crude ash (%)	1.31 ^b^ ± 0.00	1.3 ^b^ ± 0.09	1.44 ^ab^ ± 0.04	1.49 ^a^ ± 0.04	1.43 ^ab^ ± 0.01

Note: SR (survival rate); IBW (initial body weight); FBW (final body weight); WGR (weight gain rate); SGR (specific growth rate); FCR (feed conversion ratio); FI (food intake); CF (condition factor); VSI (visomatic index); HSI (hepatosomatic index). Values with different superscripts in the same row are significantly different (*p* < 0.05). A lack of superscript letters or rows with the same superscript letter indicates no significant differences between groups (*p* > 0.05).

**Table 4:** Serum biochemical indices of largemouth bass fed the five experimental diets.

Items	C	TP2	TP4	TP8	IF
TG, mmol/L	10.56 ± 1.2	9.71 ± 1.9	10.79 ± 0.9	7.95 ± 0.7	10.51 ± 1.6
CHO, mmol/L	11.86 ^a^ ± 0.2	11.12 ^ab^ ± 0.6	11.05 ^ab^ ± 0.6	10.21 ^b^ ± 0.3	11.73 ^a^ ± 0.1
HDL, mmol/L	4.94 ± 0.1	4.64 ± 0.2	4.42 ± 0.3	4.71 ± 0.1	4.92 ± 0.2
LDL, mmol/L	2.56 ^a^ ± 0.2	2.29 ^ab^ ± 0.3	2.43 ^ab^ ± 0.2	1.85 ^b^ ± 0.1	2.33 ^ab^ ± 0.2
ALT, U/L	14.01 ± 4.2	20.76 ± 1.3	17.15 ± 2.1	14.54 ± 0.7	19.33 ± 2.9
AST, U/L	83.89 ± 8.9	74.51 ± 7.9	73.46 ± 7.9	66.44 ± 5.5	71.79 ± 9.8
ACP, U/L	8.85 ^b^ ± 0.23	10.27 ^a^ ± 0.36	9.63 ^ab^ ± 0.03	8.94 ^b^ ± 0.27	9.24 ^b^ ± 0.45
ALP, U/L	251.98 ± 18.8	194.46 ± 22.3	202.5 ± 13.0	246.21 ± 12.4	232.49 ± 15.8

Note: TG (trilaurin); CHO (total cholesterol); HDL (high-density lipoprotein); LDL (low-density lipoprotein); ALT (alanine aminotransferase); AST (aspartate aminotransferase); ACP (acid phosphatase); ALP (alkaline phosphatase). Values with different superscripts in the same row are significantly different (*p* < 0.05). A lack of superscript letters or rows with the same superscript letter indicates no significant differences between groups (*p* > 0.05).

## Data Availability

The original contributions presented in the study are included in the article/Supplementary Material, further inquiries can be directed to the corresponding author.

## Supplementary Materials

The supporting information can be downloaded at the following website: https://www.mdpi.com/article/10.3390/ani15020222/s1, Figure S1. The top 20 KEGG functional difference analysis based on the Kruskal–Wallis H test.
