# Supplementary material for: Effects of Dietary Tea Polyphenols on the Growth, Antioxidant Status, Immune Function, and Intestinal Microbiota of Largemouth Bass (Micropterus salmoides)
Source: Animals (Basel). 2025 Jan 15;15(2):222. doi: 10.3390/ani15020222 (PMC11758657; doi:10.3390/ani15020222)
Supplement: Supplementary file 1 [file animals-15-00222-s001.zip › animals-3396243-supplementary.pdf]

List of supplementary Figure

Effects of Dietary Tea Polyphenols on the Growth, Antioxidant Status, Immune Function, and Intestinal Microbiota of Largemouth Bass (*Micropterus salmoides*)

Zixin Yang [1], Qiuwen Su [1], Jiafa Yang [1], Zhijun Li [1], Shanren Lan [1], Xu Jia [1], Paihuai Ouyang [2] and Huijuan Tang [1,*]

[1] College of Marine Sciences, South China Agricultural University, Guangzhou 510642, China
[2] Guangdong Weilai Biotechnology Co., Ltd., Guangzhou 511400, China
* Correspondence: tanghj@scau.edu.cn

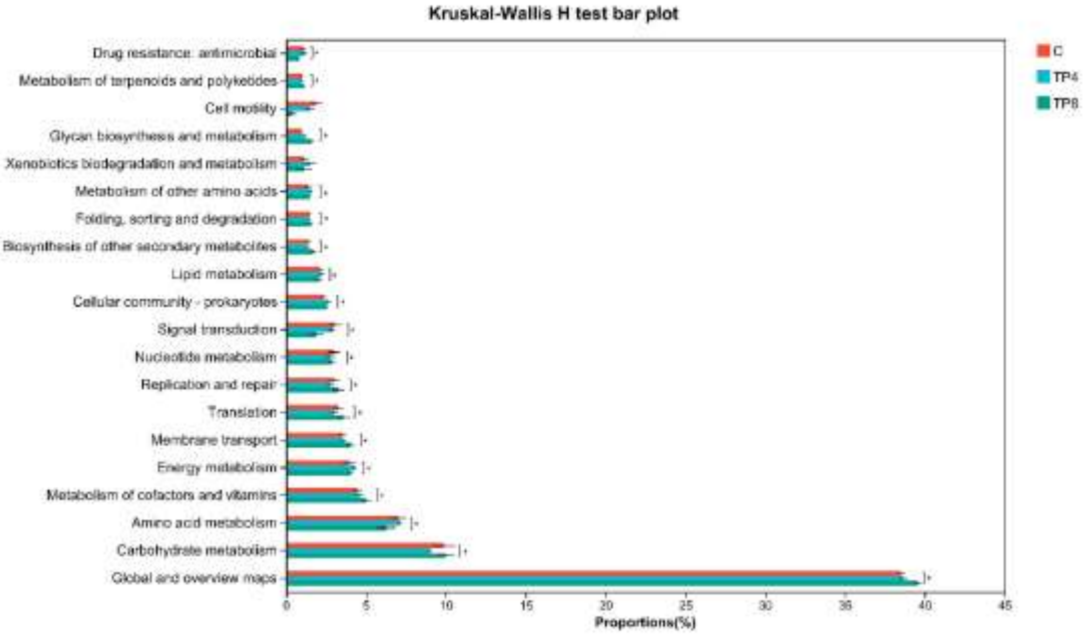

Figure S1. The top 20 KEGG functional difference analysis based on the Kruskal-Wallis H test. * $P < 0.05$.
